# Hospital and Institutional News

**Published:** 1919-09-27

**Authors:** 


### HOSPITAL AND INSTITUTIONAL NEWS.

### THE NEW SESSION.

It is more than true that with the end of the war the possibility of opening a new era in medicine is presented to the profession, and with the opening of a new session, the first which is really free from the actual disturbing influences of the world struggle, a generation of students is about to set out upon the task of bringing to maximum utility the lessons which have been so rapidly and so hardly learnt in the past few years. The one great secret of the changes war has brought is the direct effect produced by the fact that medical findings have rarely, if ever, been so severely put to test. The doctor was faced always with the direct question. His answer must always stand the test of future developments before the eyes of all. Whatever his verdict, it was sooner or later publicly proved or disproved, and so we have come more and more to study and view disease from its relation to functional efficiency. As the *Times* has recently pointed out, "the issues are clarified now, and the student will find his medicine falling naturally into three groups. First there is the prevention of disease, on which a new stress is being laid; then there is the search for cures of disease, that word being used in the sense that salvarsan is a cure for syphilis. Finally there are the means by which the natural powers of the body can be augmented, so that disease will not gain a footing or will be expelled. At the very bottom of the list comes much of the medicine of the past, the attempts to tinker up an already undermined constitution, and the treatment of symptoms where the underlying cause has passed beyond treatment."

### THE HOME WHICH THE BUILDERS REJECTED.

The winter session at the medical schools, as we note elsewhere, may be said to mark the return to normal conditions. Much has been said of the lessons learnt in the war. The time has come to prove whether the medical schools have really digested them. May not the same question be asked at this season in regard to hospital administration? There, too, lessons have been learnt, and we have now to see whether those learnt, especially in military hospitals and abroad, have been "brought home" to the minds of hospital managers in this country. A single example will suffice. Before the war much lip-service and some practical acknowledgment were paid to the value of open-air treatment. We had sanatoria for consumptives; we had country hospitals for children; we had convalescent homes for patients. But all these, except the sanatoria, were regarded as luxuries, even though the kind of luxury which no well-administered hospital could afford to be without. But the war, among other things, has conclusively proved the value of open-air treatment not merely for consumptives and surgical cases, but also for medical cases. It is a revolution in our understanding. Hitherto it has not been properly recognised in voluntary hospitals, except in so far as a few balconies are evidence of it. What is needed now is not only balconies and convalescent homes, but open-air hospitals for medical cases, so that the centre should be the convalescent homes and the periphery the hospital ward. When is this lesson to be applied generally?

### HEALTH AND THE ARMY.

A recently issued Army Council Instruction gives particulars of the new organisation of the Hygiene and Pathology Services for the Army. The main purpose is to ensure that, by co-ordination, the health and efficiency of the Army may be safeguarded by men who are recognised leaders, to utilise the most recent knowledge available, to promote economy by improving health and efficiency, by preventing wastage from disease, and by furthering research as to disease in overseas dominions. A further object is to create within the Army Medical Service an organisation which will furnish inducements to officers who have specialised in hygiene or pathology to perfect their knowledge by study and research. A number of appointments to these services at the War Office, at Home Commands, and Home Districts have been sanctioned, and will be filled up at the earliest opportunity. Appointments for commands abroad have also been provisionally approved. The duties in connection with the majority of appointments now to be filled have been carried out hitherto by specially qualified officers, so that the new arrangements will not in all cases necessarily involve new appointments.

### THE DESTRUCTION OF FLIES.

It is an old subject, but worthy of constant attention, that we should continue and maintain the anti-fly campaign throughout the country. It is taught everywhere and, indeed, by now very widely known that certain effective measures may be taken against them during the hot months; killing them, destroying their nesting grounds to render them unproductive, and all the other approved summer procedures have their percentage of value, and are undoubtedly means to be continued; but more attention might well be given to the hibernating, or "vanishing," fly, upon whose survival through the winter months the existence of millions of a new generation must depend. There is a certain amount of mystery still attaching to the process and exact locality of hibernation, but the point is that it is certain that a large proportion of the insects are within the house, out-buildings, and rubbish heaps. We read that a winter campaign is to be organised, in which the use of paint and whitewash on all interiors is to be urged, together with destruction of all autumn garden refuse. It is a practicable plan for the whole country, and most opportune at the beginning of the present post-war clearing up. It is a certain fact that if conscientiously carried out such work should materially reduce the number of flies which will influence our health and comfort next year.

### LONDON IN 1918.

The official report on the health of the Metropolis during 1918 contains the remarkable intimation that for that year, for the first time in a century's history, the deaths in London exceeded the births. Two causes contributed—the reduction in births brought about by the war and the large number of deaths from the two influenza epidemics. Among civilians alone during the year deaths outnumbered the births by nearly 5,000, and this is, of course, exclusive of the mortality among Londoners on active service. The first week in November, at the height of the second influenza outbreak, saw a death-rate of 55.5 per 1,000—the highest recorded in any week since the great cholera outbreak of 1849, when at one period the rate was 72 per 1,000! Of the civilian deaths, which were nearly 76,000 in 1918, it is probable that no fewer than 18,000 were due to influenzal infection.

### MEDICAL DEFENCE UNION.

The annual report of the Union was presented at the meeting at Leicester, under the presidency of Sir John Tweedy. One hundred and twenty-three cases were referred during the year 1919 to the Union's solicitor, twenty-four being libel and slander actions prosecuted or defended; fifteen malpraxis actions, defended; eight prosecutions of unqualified persons; seventy-six arbitrations and various. No action conducted by the Union has been lost during the last two years—an admirable record—while the profit and loss account showed a credit balance of £2,016 at the start and £2,830 at the end of the last year. The report also records the Union's difference of opinion with the General Medical Council on the subject of requests addressed by the acting registrar of the Council to members of the Union asking their reasons for refusing medical attendance upon certain cases regarding which the Council has received complaints. The report gives at length the reply of the Penal Cases Committee of the Council, justifying the Council's action. The view is upheld by the Union that if this new procedure continues, it must inevitably lead to conflict between the Council and the registered medical practitioners.

### CRUELTY TO ANIMALS.

We understand that the Home Secretary is about to appoint an inspector under the Cruelty of Animals Act of 1875. The appointment will be, in the first instance, for a period of four years, and the salary will be £800 a year, together with a war-bonus on the scale authorised for the Civil Service and travelling allowances at the usual Civil Service rate. The new post is a whole time appointment. Candidates for this post who possess the necessary scientific qualifications required for the work should apply to the Private Secretary, Home Office, London, from whom they may obtain further particulars. One or two testimonials and particulars of the candidate's qualifications should accompany the applications, and must reach the Home Office not later than October 10.

### WOMEN DOCTORS AND THE WINTER SESSION.

The new session of the medical schools, which will begin in October, may be regarded as the first virtually normal session since the war. Those who gained immense experience at the different fronts have largely returned. Those who took their places, especially the women doctors, are finding that the term of their opportunity has been reached. How far will the new plans, which are reflected in the new appointments, succeed in marrying clinical experience to teaching and research? What will happen to the women doctors now that they have lost their temporary monopoly? There must be heart-searchings, but there can be no permanent set-back. The women doctors gave general satisfaction, and that satisfaction in the long run must stand them in excellent stead notwithstanding severe competition and the traditional privilege in favour of the men. In the provinces, at all events outside university towns, the women have probably little to fear. What happens to their prospects in London is a good test of the alleged change brought about in all our notions by the war.

### THE MULTIPLICATION OF UNITED STATES HOSPITALS.

The growth of the hospital idea in the United States is shown by the recent report of the Department of Labour, which reveals several striking facts. In 1873—less than fifty years ago—there were in the United States only 149 hospitals; to-day there are nearly 9,100—an increase of nearly 6,000 per cent. in the number of institutions. The bed capacity in the first-named year was 35,453; to-day the hospitals have a total capacity of 869,000 beds—a more remarkable increase. The amount of money invested in these institutions is roughly estimated at two billion dollars, and the annual expenditure at three-quarters of a billion. This is strong testimony, considers *The Modern Hospital*, that the people of the United States are thoroughly converted to the hospital idea, and do not mean to be deprived of the benefits of hospital care. But wisdom must necessarily go hand-in-hand with progress. It must now be not so much a case of convincing people of the value of hospitals as of teaching them the necessity of measuring the needs of the community before building or even planning a hospital.

### DEATH OF THE CHEMIST TO THE KING.

By the death of Sir Peter Wyatt Squire pharmacy loses a distinguished member who has done much for the advancement of the scientific side of chemistry. Sir Peter was chemist and druggist to His Majesty King George, having held the same position to the late King Edward and also to Queen Victoria. "Squire's Companion" to the British Pharmacopœia, well known to all pharmacists and to many medical men and students, was compiled by Sir Peter Squire. It is a standard commentary on the British Pharmacopœia, and as a guide to "unofficial" preparations it still holds a high position among pharmaceutical text-books. The funeral, which took place on the 19th inst., was attended by prominent pharmacists and many medical men who had frequently consulted with Sir Peter Squire for the sake of his probably unrivalled knowledge.

### THE MINISTRY OF HEALTH AND TUBERCULOSIS OFFICERS.

On June 6, the Lambeth Borough Council appointed Dr. Philip Smith Tuberculosis Officer at the Central Tuberculosis Dispensary, subject to the approval of the Local Government Board, now the Ministry of Health. Although over three months have elapsed, the approval has not been received. This long delay in approving his appointment has compelled Dr. Smith to withdraw. The council has decided to express regret to Dr. Smith, and to the Ministry of Health. As over three months have gone by since his appointment, and some of the candidates who were not then in a position to apply may now desire to do so, the council has agreed to a proposal of the Public Health Committee at once to advertise the vacant position so that they may select another Tuberculosis Officer at their meeting on October 16.

### HOUSING AT NOTTINGHAM.

An echo of the unsatisfactory understanding which appears to exist between local authorities and the Government on the question of housing schemes was heard at a recent meeting of the Nottingham City Council, when extensive schemes previously decided upon for the provision of new houses had to be rescinded. It was contended that the cost involved would be altogether disproportionate to the rents received from the tenants, while there was nothing under the Government arrangements which would safeguard the municipality against heavy loss ultimately. The alternative which was approved appears poor by comparison. The Housing Committee was instructed to prepare projects for providing necessary accommodation by the erection of a reasonable number of houses, or tenement dwellings, in suitable positions where streets were already made and sewers and gas mains laid. We have not exact details to hand, but the whole sounds very much like forced falling back from the great ideal of modernisation and fresh air, a more than disappointing tendency after we have heard so much, a few months ago, of the refinement and freedom from crowding and the old ideas, with which the new homes of the country were to be erected everywhere.

### "AN ABUNDANCE OF HOSPITAL BEDS."

Though the lay Press did its best to make Sir E. Napier Burnett, K.B.E., M.D., Chairman of the North-Eastern Hospitals' Association, appear a Jeremiah, the real point which he made was a constructive one. Like the hospital men of Birmingham and Bristol, Sir Napier Burnett is in favour of closer co-ordination among voluntary hospitals, as all must be who know the facts and also how to make the best of them. On behalf of the North-Eastern Hospitals' Association, Sir Napier Burnett has lately served the district from Berwick-on-Tweed to Middlesbrough. He has found, he told the *Daily Chronicle*, not, be it noted, merely hospitals with long "waiting-lists of patients needlessly suffering and needlessly detained," but, which is at least of equal importance, "an abundance of hospital beds in the area if only they were properly co-ordinated." The speaker is one who has been trained to weigh his words, and since he has found "an abundance of hospital beds" but a want of co-ordination, is not the news exceedingly encouraging? Time and money are needed for the building of new wards; more money to maintain them. But for co-ordination only two simple things are needed—namely, tact and brains. As to the financial bugbear, which Sir Napier did not unduly exploit, the voluntary system has survived difficulties at least equal to the present. Despair condemns its victims. What we need here, too, is tact in brains—in other words, men who believe in the voluntary system sincerely enough to communicate their belief to others. The catchpenny clerk cannot save us. The men of conviction will.

### A SANITARY SURVEY OF THE NATION.

In an address on hospital organisation and management delivered by Sir Napier Burnett at the Aberdeen Royal Infirmary, an appeal was made for a sanitary survey of the nation, with a view to mapping out the land for the purpose of establishing local authorities which would unify and direct all local energies pertaining to health. Such survey had been carried out by the North-Eastern Hospitals Associations in an area of about 4,000 square miles in the North of England and had afforded important data. The shortage of hospital beds had an important bearing on the question of national economy by way of unemployment and lessened production, and was also a causal factor in the general social unrest. If the voluntary system could not put the matter right the Ministry of Health would have to supply the accommodation. In reference to hospital finance, Sir Napier Burnett expressed the hope that the day was not far distant when King Edward's Hospital Fund, or some Government subsidy, would extend its operations to Provincial hospitals, coupling with the aid given some co-ordinated system of central control. The need of hospital accommodation for middle-class patients who were able to pay one, two, or three guineas a week for a bed in a general hospital, called for very serious consideration. The day would appear to be approaching when hospital managers would have to consider the payment of a retaining fee to the medical men on the staff. Each surgeon and physician on the staff ought to have a number of medical practitioners associated with him in his hospital duties.

### A STANDARD FOR HOSPITAL PRESENTATIONS.

Mr. E. J. Domville, resident senior house surgeon to the Devon and Exeter Hospital, has been presented with a grandfather clock and an illuminated address by Sir Chaning Wills on behalf of the staff for his patriotic service during the war. In congratulating the donors and the recipient on the presentation, we hope that the illuminated address was worthy of them both. This presentation happened to coincide with our plea in The Hospital of the 13th instant for the entrusting of such documents to the pens of craftsmen of repute. Lettering and illumination are being exquisitely practised by certain well-known scribes to-day, as the recent War Memorial Exhibition at South Kensington showed, and therefore we follow our reference to the lesson of that exhibition by repeating the question which it raises in connection with the next presentation of the kind that we have observed. It is perhaps noteworthy that while the name of the clockmaker has been published, we have seen no mention of the craftsman who wrote the address. The point is that the hospital standard should apply to everything in institutional life, including the arts which are employed on these occasions; and now that the art of lettering flourishes, and that its masters' names are known, there is no excuse for the hospitals not to have the best artistic value for their money.

### THE PROSPECTS OF THE WEST KENT HOSPITAL.

With general goodwill the two beds provided by the people of Town Malling and district at the West Kent General Hospital, Maidstone, in memory of the fallen, were ceremoniously "unveiled" last week. Colonel Sir Charles E. Warde, Bart., accompanied by Mr. Gerald Mercer and Mr. B. P. Boorman, representing the Governors, received the visitors. The two beds thus provided reflect a spirit which may gain its ambition, namely, to establish a hospital with 100 beds. For this reason the occasion was significant. It is also inspiring to record that the prime movers in the matter include the commandant of the Malling V.A.D. Hospital, Mrs. Wingfield Stratford, a member of whose family, by the way, is the author of an encyclopædic "History of English Patriotism." Since the future of the voluntary hospitals depends largely on continuance of the active interest formed during the war between those who became V.A.D.s and their families and the hospitals, it is really gratifying to note how this welcome assistance to the West Kent General Hospital came about.

### A COMPARISON FOR CARDIFF.

A welcome and gratifying gift has been received at King Edward VII. Hospital, Cardiff, in the form of a cheque for 1,400 francs from Colonel C. E. R. Isherwood, on behalf of Lieut.-General Sir Beauvoir de Lisle, K.C.B. The latter commanded the 15th Corps, whose officers, non-commissioned officers, and men, on their demobilisation, desire to record their gratitude by this present. They wish to recognise "the many valuable services" rendered by the hospital's staff "to soldiers and their dependants during the war." We take it that the generous donors have become recruits to the voluntary system, and their gift shows a spirit which, alas! is sometimes lacking in hospital men who, at the first sign of difficulty, confess their own want of courage, and raise the absurd cry of hospital "bankruptcy." Mr. Leonard Rea, the general superintendent at Cardiff, is far too good a man not to welcome the spirit of the donors of this gift. How gladly he would find it, if he could, among the civilians of Cardiff whose friends and dependants *likewise* benefit by the services of King Edward VII. Hospital. He may well look round on the huge local population served by the hospital and say, "Were there not ten cleansed, but where are the nine?" Is gratitude dead in every breast except that of the soldier?

### THE FINAL ACT OF THE ENFIELD HOSPITAL SCANDAL.

The position of affairs at the Enfield Hospital has reached the *reductio ad absurdum* which we predicted when the medical staff first attempted to govern the situation without respect to the Committee. The chief officers in consequence resigned, the hospital is now without a Secretary, a Treasurer, and an Executive Committee, and the trustees last week passed the following resolution:—

> "That the Trustees, having reluctantly come to the decision that unless new officials and a new Council can be appointed, and the necessary funds provided for carrying on the hospital, it will be necessary to close the hospital, therefore give notice that the hospital will be closed after December next, unless satisfactory arrangements can be made in the meantime."

In consequence of this decision, which was inevitable, the Chairman of the Enfield Urban District Council placed on the agenda of the Council at the last meeting a notice of motion, the effect of which was to request the Ministry of Health to institute a Public Inquiry, and to authorise the Urban District Council to take the necessary steps to provide the hospital accommodation of which the population of the district, numbering 60,000, has been deprived.

### THIS WEEK'S DRUG MARKET.

Business is fairly good, but without any outstanding features of first-rate importance. Price fluctuations are both upwards and downwards, there being about as many "downs" as "ups." It is therefore clear that the long-hoped-for general fall in prices has not yet come. Senna leaves are again dearer. Rhubarb is still scarce, and fetches extreme prices. Ergot of rye is still in very short supply and very dear. Clove oil has again moved upwards in price, in consequence of the further rise in raw material. Saffron, sarsaparilla, Japanese dementholised peppermint oil and Japanese refined camphor are also dearer. On the other hand, lemon oil and bergamot oil have a downward price tendency. Phenacetin, phenazone and hexamine are in rather better supply.

